# Supplementary material for: Toughening colloidal gels using rough building blocks
Source: Nat Commun. 2023 Aug 31;14:5309. doi: 10.1038/s41467-023-41098-9 (PMC10471594; doi:10.1038/s41467-023-41098-9)
Supplement: Supplementary file 1 — Supplementary information [file 41467_2023_41098_MOESM1_ESM.pdf]

Supplementary Information: Toughening colloidal gels using rough building blocks.

Florence J. Müller[1], Lucio Isa[1], and Jan Vermant[1]

[1]Department of Materials, ETH Zurich, Switzerland.

1 Thermoreversible model system synthesis

We developed a two-step grafting method to reproducibly bind octadecyl to the surface of rough and smooth silica particles at comparable coverages (see Figure 1a. and methods, Figure SI 1). To this end, smooth SiO_2 core particles (Figure SI 2a. and b.) are synthesized using the Stöber method [1]. Rough particles (Figure SI 2c. and d.) are assembled using the electrostatically-driven heteroaggregation protocol previously described by Zanini et al. [2, 3], where negatively charged asperity particles are adsorbed to the surface of a positively charged core particle. The rough "raspberry" assembly is then stabilized and functionalized by the hydrolysis and condensation of the secondary amine silane (Trimethoxy[3-(methylamino)propyl]silane (MAPTMS)) - the smooth particles are functionalized by the same process. In parallel, octadecanol is functionalized with an alkynoate group, using the Fischer esterification process. This high-yield reaction can be performed on a 10 g scale or larger. Finally, the octadecane-alkynoate and the secondary-amine-functionalized silica particles can be mixed in isopropanol at 40°C, in order for the click-like reaction to take place without catalyst or initiator (see Figure SI 1). This reaction is highly efficient, which allows for high octadecyl surface coverage as needed for the thermoreversible attraction between the particles. Figure SI 2 shows SEM images of the octadecyl-coated silica particles with smooth (Figure SI 2 a. and b.) and rough (Figure SI 2 c. and d.) cores, as well as AFM data for smooth and rough particles (Figure SI 2 c. and f., respectively). For the characterization detailed in the main text, the hydrodynamic radii of both rough and smooth particles are of 300 nm. Both, rough and smooth particles, can be dried and re-suspended in tetradecane (C_{14}), to form a suspensions which can be thermoreversibly gelled (SI figure 4). As shown in Figure 1b., at high temperature (25-60°C) the octadecyl (C_{18}) forms a steric repulsion layer around the particles. When the system is cooled (5-20°C), the octadecyl brush crystalizes with the tetradecane and enable adhesive van der Waals forces, leading to a particle network formation [4, 5].

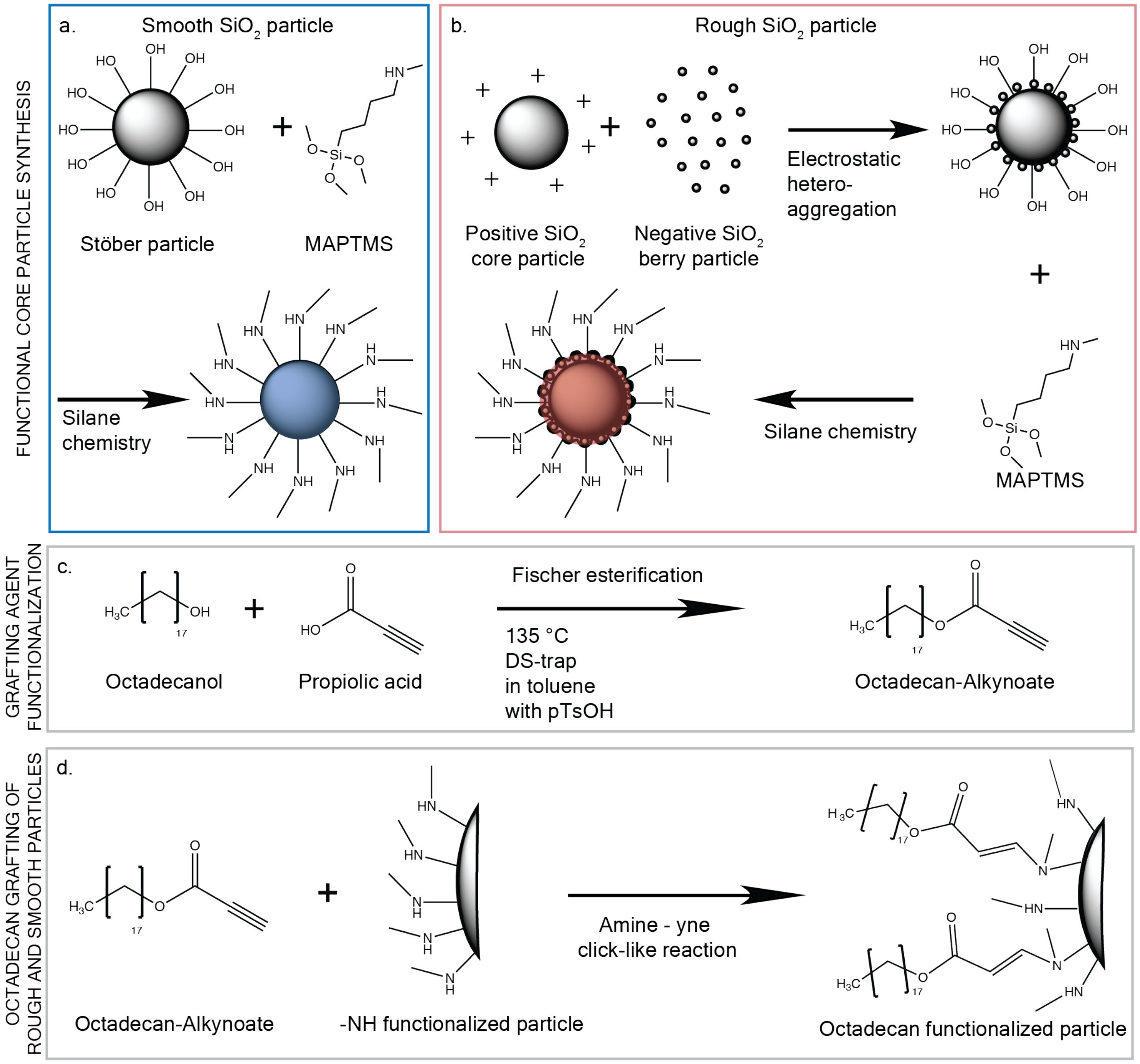

Figure S1: Complete synthesis scheme for smooth and rough octadecyl-functionalized particles. a. Synthesis of smooth Stöber particles and surface grafting of secondary amine groups. b. Electrostatic heteroaggregation of negatively charged SiO_2 asperity particles to positively charged SiO_2 core particles with subsequent stabilization and functionalization with MAPTMS, c. Fischer esterification of octadecanol with propiolic acid for the synthesis of the functional grafting agent Octadecan-alkynoate, d. Click-like reaction of Octadecan-alkynoate onto the surface secondary amine groups on smooth and rough particles.

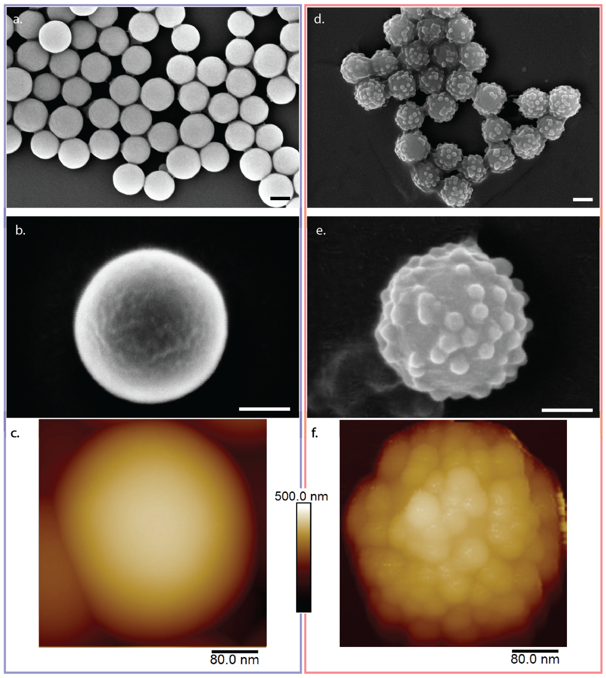

Figure S2: Characterization of smooth and rough particles. a.,b. SEM image of smooth particles (scale bar 200 nm and 100 nm, respectively), c. AFM of smooth particle, d.,e. SEM image of rough particles (scale bar 200 nm and 100 nm, respectively), e. AFM of rough particle.

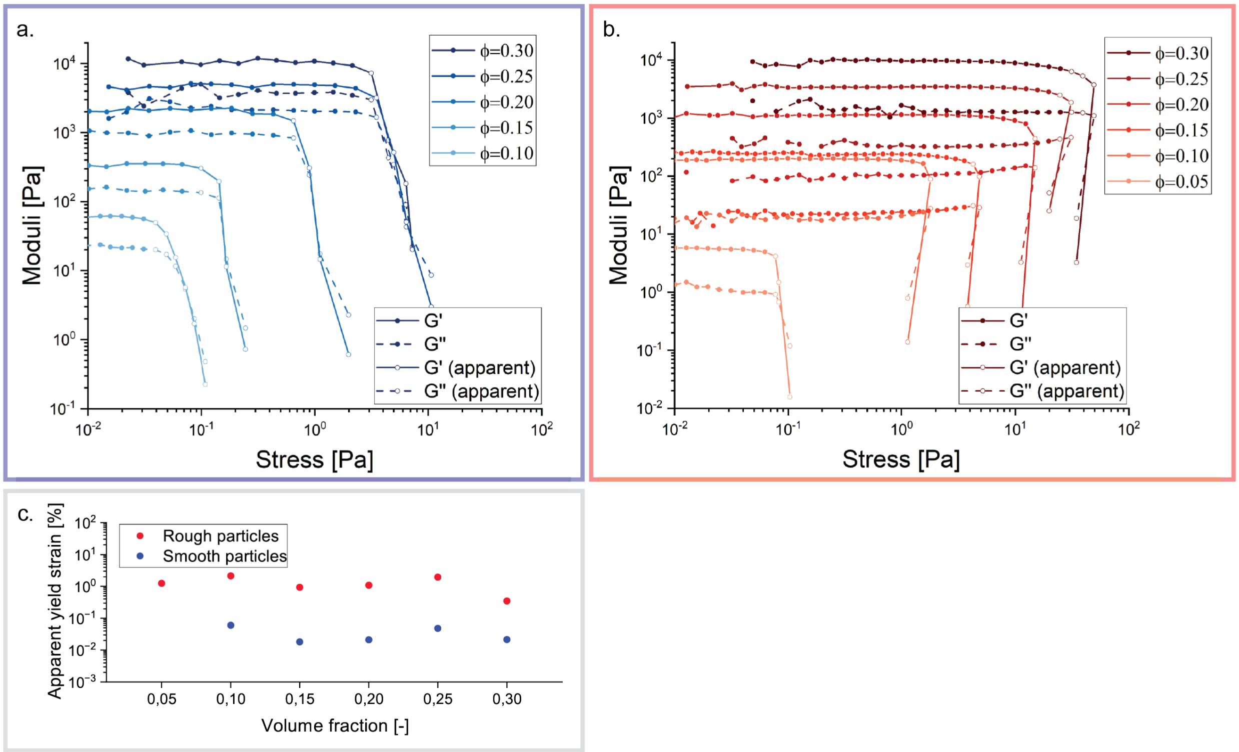

Figure S3: Stress amplitude sweep for a. Rough, and b. Smooth particle gels at varying volume fraction (the solid symbols represent reliable data and the empty symbols represent data points that might be affected by measurement errors, see main text) c. Apparent yield strain for rough and smooth particle gels at different volume fractions.

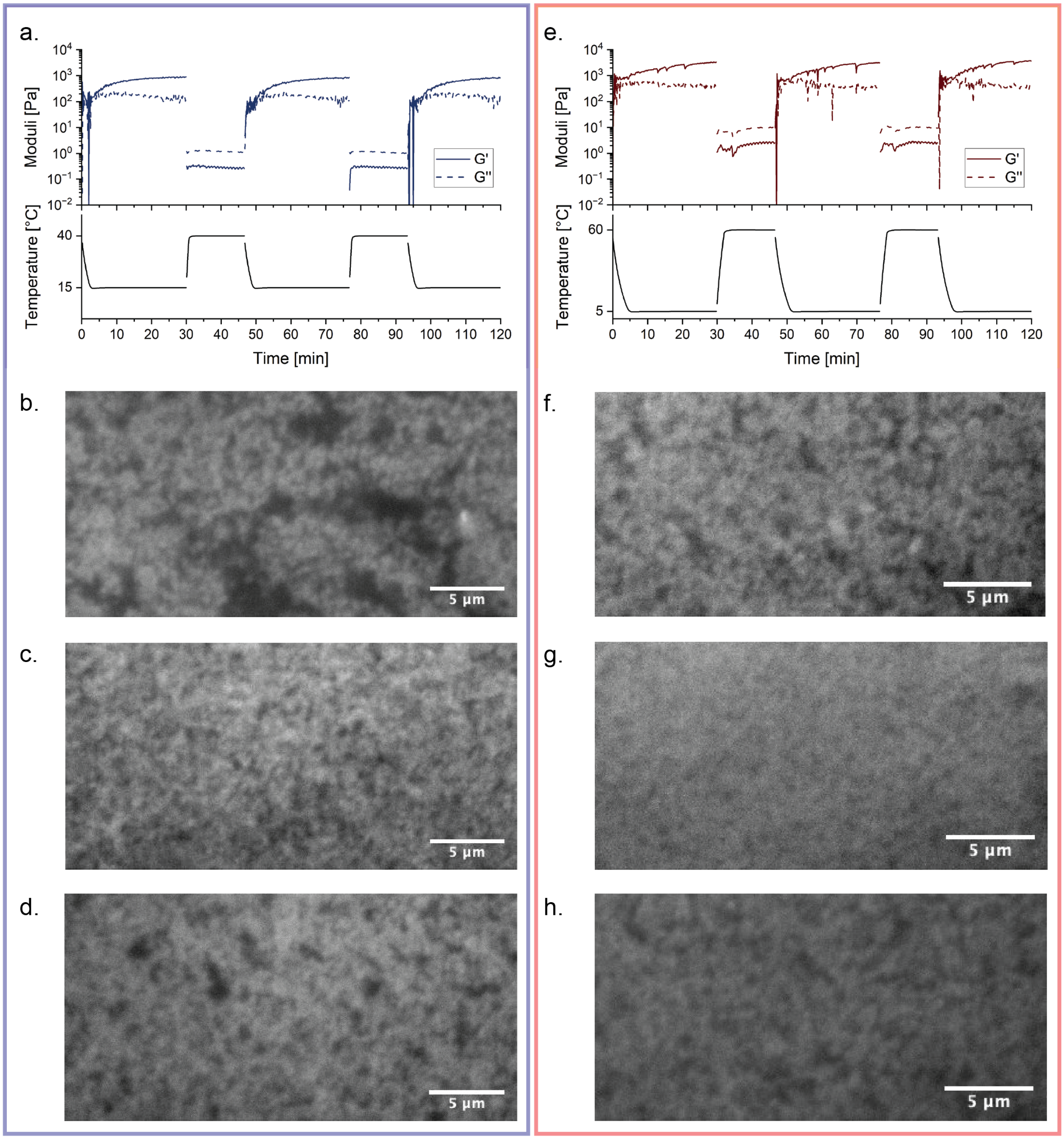

Figure S4: Temperature response of the smooth (blue, left) and rough (red, right) particle gels (ϕ_v=0.25). a. Temperature cycle of smooth particle gel at volume fraction ϕ=0.25, b. Confocal image of a smooth particle gel after loading using a pipette at 5 °C, c. Confocal image of a smooth particle gel at 60 °C, d. Confocal image of a smooth particle gel at 5 °C after heating to 60 °C, e. Temperature cycle of rough particle gel at volume fraction ϕ=0.25, f. Confocal image of a rough particle gel after loading using a pipette at 5 °C, g. Confocal image of a rough particle gel at 60 °C, h. Confocal image of a rough particle gel at 5 °C after heating to 60 °C.

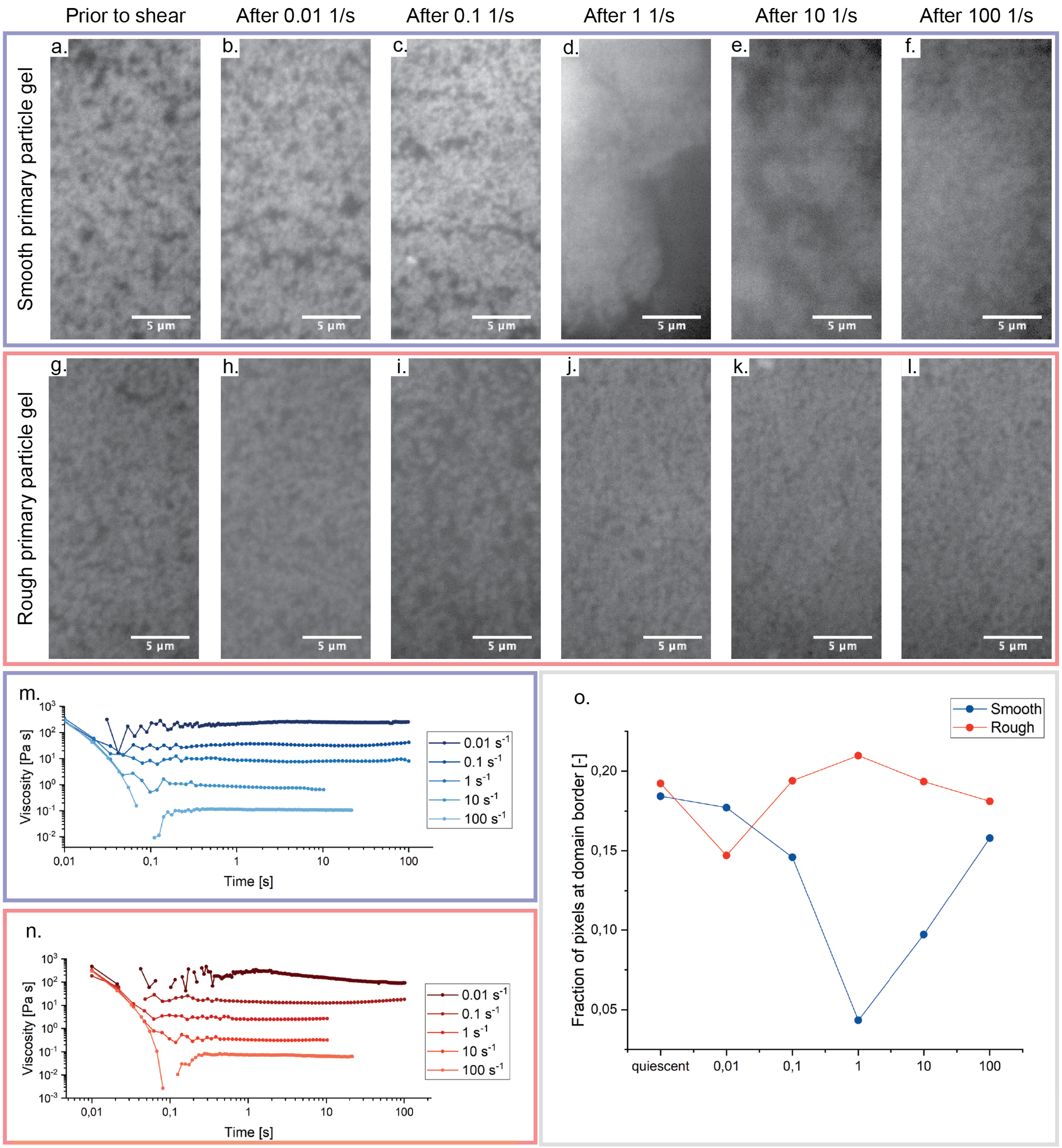

Figure S5: a.-f. microstructure of smooth particle gel (ϕ_v=0.25), a. After quiescent gelation, b. After shearing at 0.01 1/s, c. After shearing at 0.1 1/s, d. After shearing at 1 1/s, e. After shearing at 10 1/s f. After shearing at 100 1/s g.-l. Microstructure of rough particle gel, g. After quiescent gelation, h. After shearing at 0.01 1/s, i. After shearing at 0.1 1/s, j. After shearing at 1 1/s, k. After shearing at 10 1/s, l. After shearing at 100 1/s, m. Flow curves corresponding to the images in images a.-f., n. Flow curves corresponding to the images in images g.-l, o. Moran's Index analysis of the images a.-l. (see section SI 2).

2 Moran's Index calculation

The confocal data shown in figure S5 were quantitatively compared using the spatial autocorrelation, the so called Moran's index (Moran's I). To this end, the images were first binarized using the thresholding function on ImageJ. The local Moran's I was calculated for every pixel through the moransI2 function in Matlab (https://ch.mathworks.com/ matlabcentral/fileexchange/13663-moran-s-i) using a 5x5 window. Here, the Moran's I can have values between 0 and 1, 0 being a 0-pixel (black) surrounded by 0-pixels, and 1 being a 1-pixel (white) surrounded by 1-pixels. Intermediate values represent the case of either black or white pixels surrounded by a mixture of 0- and 1-pixels. In this analysis, we computed the local Moran's I for every pixel, and excluded the pixels with Moran's I equal to 1 and 0, because they belong to fully white (interior of the gel) or fully black (background) regions, focusing on the intermediate values of Moran's I, which represent the boundary between the gel and the background. The values of I for intermediate pixels were added and divided by the total amount of pixels in the image, which is represented in figure S5. For instance, for the smooth case, after shearing at 1 s^{-1}, the fraction of pixels at the domain border is lowest as there is a clear phase separation observable in figure S5d.. Moreover, the values of I for rough and smooth particle gels in the quiescent states and low shear rate states is very similar, which can also be qualitatively seen in the corresponding images.

References

[1] W. Stöber, A. Fink, and E. Bohn, “Controlled growth of monodisperse silica spheres in the micron size range,” *Journal of Colloid and Interface Science*, vol. 26, 1968.

[2] M. Zanini, C.-P. Hsu, T. Magrini, E. Marini, and L. Isa, “Fabrication of rough colloids by heteroaggregation,” *Colloids and Surfaces A: Physicochemical and Engineering Aspects*, vol. 532, pp. 116–124, 2017, ISSN: 09277757. DOI: `10.1016/j.colsurfa.2017.05.084`.

[3] K. Furusawa and C. Anzai, “Preparation of composite fine particles by heterocoagulation,” *Colloid and Polymer Science*, vol. 265, pp. 882–888, 1987.

[4] A. P. Eberle, N. J. Wagner, B. Akgun, and S. K. Satija, “Temperature-dependent nanostructure of an end-tethered octadecane brush in tetradecane and nanoparticle phase behavior,” *Langmuir*, vol. 26, no. 5, pp. 3003–3007, 2010.

[5] S. Roke, J. Buitenhuis, J. Van Miltenburg, M. Bonn, and A. van Blaaderen, “Interface–solvent effects during colloidal phase transitions,” *Journal of Physics: Condensed Matter*, vol. 17, no. 45, S3469, 2005.
